# Perceived and Actual Social Discrimination: The Case of Overweight and Social Inclusion

**DOI:** 10.3389/fpsyg.2013.00147

**Published:** 2013-04-01

**Authors:** Freda-Marie Hartung, Britta Renner

**Affiliations:** ^1^Psychological Assessment & Health Psychology, Department of Psychology, University of KonstanzKonstanz, Germany

**Keywords:** perceived discrimination, actual discrimination, accuracy, social inclusion, overweight, BMI, gossip

## Abstract

The present study examined the correspondence between perceived and actual social discrimination of overweight people. In total, 77 first-year students provided self-ratings about their height, weight, and perceived social inclusion. To capture actual social inclusion, each participant nominated those fellow students (a) she/he likes and dislikes and (b) about whom she/he is likely to hear social news. Students with lower Body Mass Index (BMI) felt socially included, irrespective of their actual social inclusion. In contrast, students with higher BMI felt socially included depending on the degree of their actual social inclusion. Specifically, their felt social inclusion accurately reflected whether they were actually liked/disliked, but only when they were part of social news. When not part of social news, they also showed insensitivity to their actual social inclusion status. Thus, students with a lower BMI tended to be insensitive, while students with a higher BMI showed a differential sensitivity to actual social discrimination.

## Introduction

More than 1.4 billion adults, 20 and older, were overweight in 2008 (World Health Organization, 2012). Today, overweight and obese individuals face discrimination in almost every domain of living (cf. Puhl and Heuer, 2009; Schupp and Renner, 2011). Specifically, discrimination is pervasive in the social domain (e.g., not being included in social groups, others talking negatively about obese individuals; Sitton and Blanchard, 1995; Hebl and Xu, 2001; Puhl and Brownell, 2006), the economic domain (e.g., having difficulty in getting hired; cf. Carr and Friedman, 2005; Fikkan and Rothblum, 2005), and the physical domain (e.g., receiving less careful treatment from physicians; Hebl and Xu, 2001; Carr and Friedman, 2005). In contrast to widely recognized social stigmata, such as race or gender, there are no legal sanctions in place to protect individuals from obeseism. This fact is of particular concern as the prevalence of perceived obeseism has increased in recent years, and the growth is only partly explained by changes in obesity rates (Andreyeva et al., 2008). Thus, the degree of actual and perceived obeseism is of vital theoretical and practical interest.

In general, one way to investigate discrimination is to focus on the source of discrimination by capturing the perspective of the stigmatizing individual. In the realm of social discrimination, physicians reported that they would spend less interaction time with overweight patients than with normal weight patients (Hebl and Xu, 2001), and Sitton and Blanchard (1995) showed that fewer men responded to ostensible lonely hearts ad when the woman was described as overweight than when the woman had a history of drug addiction. That is, these studies assess the extent of self-reported or actual observed discrimination toward overweight individuals.

Other studies capture the perspective of the stigmatized individual (target of discrimination; e.g., Carr and Friedman, 2005). Puhl and Brownell (2006), for example, asked members of a weight loss support group organization to report the frequency of discrimination. They found that about 50% of the participants believed that they had already experienced social discrimination such as “being avoided, excluded, ignored” at least once in their life time. That is, these studies assess the perceived discrimination by the target of discrimination.

When considering both sources and targets of discrimination, the question arises whether and to what extent actual and perceived discrimination effectively corresponds. Theoretically, potential targets of discrimination can perceive discrimination when it occurs (accurate perception), fail to perceive it (insensitive perception), or even perceive discrimination that has not occurred (oversensitive perception; cf. Feldman Barrett and Swim, 1998; Major et al., 2002). Supporting the notion of oversensitive perception, research has shown that former experience of discrimination leads to expectations of future discrimination. In turn, expecting to be discriminated against may trigger a more ready perception of ambiguous behavior as discriminatory (Feldman Barrett and Swim, 1998; Major et al., 2002). Especially impressive are findings showing that stereotypes may even affect stigmatized individuals in the absence of actual discriminatory behavior (e.g., Steele and Aronson, 1995). Thus, perceived discrimination may indicate actual discrimination or may reflect systematic over- or underestimation of actual discrimination.

The central aim of the present study was to examine the correspondence between perceived and actual social discrimination. To study social discrimination from both the target and source perspectives, we examined perceived and actual social inclusion within a newly forming social network of first-year students in a real life setting. In order to assess the degree of actual social discrimination toward overweight fellow students, we examined the relation between actual social inclusion [i.e., peer rated social preference of and gossip activity about the respective target person] and Body Mass Index (BMI) of the target person. Likewise, to assess the degree of perceived social discrimination of overweight students, we examined the relation between individuals’ perceived social inclusion (i.e., self-rated social inclusion) and BMI. Moreover, to assess the accuracy of perceived discrimination relative to one’s body weight, the correspondence between actual social inclusion and perceived social inclusion was analyzed with relation to the subjects’ bodyweight.

## Materials and Methods

### Procedure

The present data were collected within a greater research project examining the antecedents and consequences of network formation and consolidation in a freshmen sample [Social Network Study (SozNet)]. Participants were psychology first-year students at the University of Konstanz, Germany. They were informed about the study and invited both during the introduction week and by email to participate in a study on “Social Networks.” The first measurement took place 1 week after the beginning of the semester. Participants filled in an online questionnaire every 2 weeks throughout their first semester providing information about themselves and their relationship to other first-year students. Data from the second measurement is missing due to technical problems during data collection. Thus, nine measurement points are available. As compensation for their participation participants received a 20 € book voucher, up to 5 h of course credit, and feedback on the study results.

### Participants

The sample comprised *N* = 77 students (*n* = 62 female, 80.5%) with a mean age of 22.37 years (17–47 years, SD = 5.96). Missing values were estimated using multiple imputation (MI, SPSS 20, cf. Schafer and Graham, 2002).

### Measures

All variables were assessed at all nine points of measurement unless otherwise indicated.

#### Body Mass Index

At T1, participants were asked to report their height and weight. On average, participants had normal weight with a mean BMI (weight in kg/height in m^2^) of 22.31 (SD = 3.12), ranging from 17.78 to 34.57.

#### Perceived social inclusion

Perceived social inclusion was assessed through five questions (i.e., Williams et al., 2000; Eisenberger et al., 2007). In particular, participants were asked to rate their perceived degree of social inclusion while thinking about their fellow students from the previous week (for instance “Thinking of your fellow students last week, how much did you … … belong?, … feel integrated?”). Ratings were provided on 7-point rating scales (1 not at all–7 very much). On average, participants reported high social inclusion across the nine points of measurements (*M* = 5.46, SD = 0.95–1.06; α = 0.90).

#### Actual social inclusion

Actual social inclusion was assessed using two aspects of social status: *social preference* and *gossip activity about target*. Both aspects were measured through nomination procedures, where participants received a complete roster of fellow student names alphabetized by first name.

##### Social preference

Participants nominated the three fellow students they “like most” (LM) and the three fellow students they “like least” (LL). LM- and LL-scores were computed for each participant by counting the nominations received for LM and LL nominations, respectively. Across all nine points of measurement, each participant was named on average 20 times (SD = 12.60) as most liked and 15 times (SD = 31.22) as least liked. LM- and LL-scores were standardized for each measurement point and used to generate social preference scores by calculating LM–LL (cf. Coie et al., 1982). Thus, the social preference-score reflects the *likeability* of students (Cillessen and Rose, 2005).

##### Gossip activity

Gossip is conceptualized as conversation about social and personal topics (Dunbar, 2004). To capture the gossip activity about a particular student, participants nominated those three fellow students about whom they would most likely hear interesting social news (“Über welche Person aus Ihrem Semester würden Sie am ehesten interessante Neuigkeiten erfahren?”; cf. Foster, 2003; McAndrew et al., 2007). Gossip activity scores were computed for each participant by counting the nominations received per point of measurement. On average, each participant was nominated 17 times (SD = 30.81) across the nine measurements. Thus, the gossip activity score reflects how much fellow students talked about a particular student.

#### Statistical analyses

Since observations were nested within persons, the data structure is hierarchical. Therefore, we used multilevel modeling (SPSS 20) to allow an assessment of average effects across individuals (fixed effects), taking into account that individuals vary around this average value (random intercept and slopes). Specifically, a two-level hierarchical model was calculated that assessed the fixed effects of social preference, gossip activity, BMI, and the respective interaction terms on perceived social inclusion. To control for time trends *time* was included as a predictor in the model. As we were interested in individual differences, both level 1 predictors (gossip activity and social preference) and level 2 predictors (BMI) were grand-mean centered. *Time* was centered at the first measurement point. To control for variability between individuals, the intercept and the slope of *time* were permitted to be random.

Multilevel modeling procedures of SPSS 20 support MI datasets and pool relevant statistics (e.g., *b*s and *p*s). However, with multilevel modeling *df* are not pooled. Therefore, the range of *df* for the multiple datasets is reported. To follow-up significant interaction terms and test simple slopes, we used a tool provided by Preacher et al. (2006).

## Results

### The two perspectives of social discrimination: Perceived and actual social inclusion

In a first step, bivariate relationships between BMI and the two perspectives of social discrimination were assessed. BMI and mean perceived social inclusion were negatively correlated (*r* = −0.29, *p* = 0.01), showing that individuals with a higher BMI perceived themselves as being less socially included. Conversely, BMI and social preference were not significantly correlated (*r* = 0.10, *p* = 0.40), indicating that students with a higher BMI were neither less nor more liked by their fellow students. However, BMI and gossip activity (*r* = −0.29, *p* = 0.01) were negatively correlated, indicating that fellow students gossiped less about students with a higher BMI.

### Match between perspectives

In the next step, correspondence between perceived and actual social inclusion was assessed. Therefore, multilevel model analysis was conducted with perceived social inclusion as the dependent variable, and BMI, social preference, gossip activity, and the respective interaction terms (see Table 1) were included as independent variables. *Time* was included as an additional variable to control for potential time effects. Fixed effects and random effects are displayed in Table 1. Both random intercept and random time slope reached significance, indicating a substantial variation between individuals in their basic degree of perceived social inclusion and their change of perceived social inclusion across time.

**Table 1:** **Fixed and random effects of multilevel analysis testing of the effect of BMI, social preference, gossip activity, and their respective interaction terms (IVs) on perceived social inclusion (DV)**.

Fixed effects	*b*	*t*-value	*df*-value
Time	−0.01	−0.41	77.12–78.14
BMI	−0.12	−3.38***	85.44–88.51
Social preference	0.13	2.55**	566.76–654.53
Gossip activity	0.02	0.78	642.18–682.15
Social preference × gossip activity	0.03	2.40*	652.12–687.73
BMI × gossip activity	0.00	0.27	634.82–680.99
Social preference × BMI	0.01	0.52	659.56–687.13
BMI × social preference × gossip activity	0.01	2.30*	586.37–615.30

**Random effects**	***b***	**Wald *Z***	

Intercepts	0.63	3.95***	
Slope (time)	0.01	2.91**	
Covariance of intercept and slope	0.00	−0.24	

As SPSS does not pool *df* of the multiple imputation datasets within multilevel modeling, the range of *df* is reported. ***p < 0.001; **p < 0.01; *p < 0.05.

**Figure 1:**
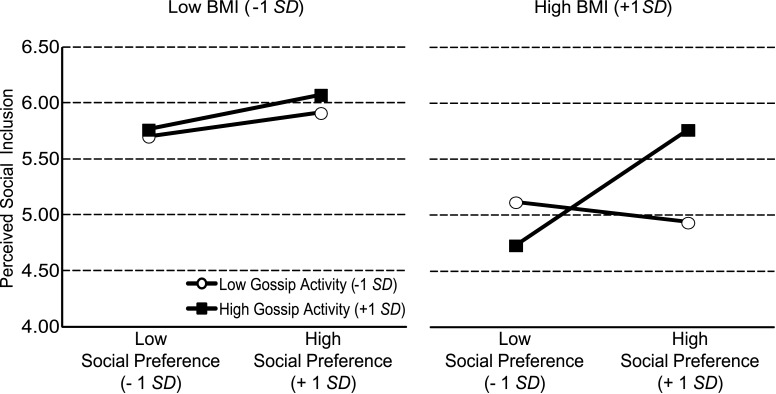
**Relationship between perceived social inclusion, Body Mass Index (BMI), social preference, and gossip activity**.

Multilevel analysis revealed significant main effects for BMI and social preference and a significant two-way interaction (see Table 1). However, these effects were qualified by a significant three-way interaction (BMI × social preference × gossip activity; see Figure 1). Simple slope analysis revealed that students with a lower BMI felt generally socially included irrespective of whether they were liked by their fellow students or not. This is true for students that were highly gossiped about (simple slope for *gossip activity* + 1 SD: *b* = 0.12, *z* = 1.13, *p* = 0.26) as well as for students who were less gossiped about (simple slope for *gossip activity* − 1 SD: *b* = 0.08, *z* = 0.77, *p* = 0.44). Thus, students with a lower BMI appeared to be comparably insensitive to actual social inclusion or exclusion.

In contrast, students with a higher BMI showed clear evidence for differential perceptions depending on the degree of actual social inclusion. Only when they were highly gossiped about were they also sensitive toward actual social preference (simple slope for *gossip activity* + 1 SD: *b* = 0.38, *z* = 0.3.74, *p* < 0.001). When they were less gossiped about perceived social inclusion was insensitive to actual social preference (simple slope for *gossip activity* − 1 SD: *b* = −0.07, *z* = −0.66, *p* = 0.51). Thus, the perceived social inclusion of students with a higher BMI depended on both the actual social preference and gossip activity about them.

## Discussion

In the present study social discrimination was examined from both target and source perspectives. Focusing solely on the perspective of the target of discrimination suggests that individuals with higher BMI experience more discrimination than individuals with a lower BMI in a newly forming social network. Switching to the perspective of the source of discrimination shows that students like fellow students with higher BMI equally well as those with lower BMI. Moreover, they even gossip less about those with a higher BMI. Thus, focusing on either perceived or actual discrimination could lead to fundamentally different conclusions about social discrimination against overweight individuals.

More importantly, broadening the view and considering the correspondence between perceived and actual social discrimination shows startling differences between individuals with higher and lower BMI: individuals with a lower BMI feel socially included, irrespective of the actual social inclusion status. In contrast, the perceived social inclusion of individuals with higher BMI depends on the actual social preference status and gossip activity about them. Interestingly, only when they were part of social news did they accurately perceived social discrimination toward them: they felt more socially included when they were highly liked and less included when less liked by others. Thus, individuals with lower and higher BMI show differential accuracy in their perception of social discrimination. Whereas students with a lower BMI tended to be insensitive to actual social discrimination and were positively biased, students with a higher BMI showed a higher sensitivity and tended to be negatively biased.

This pattern of results suggests that the differences in perceived discrimination by individuals with lower BMI as compared to individuals with higher BMI are not solely due to differences in actual discrimination (e.g., Feldman Barrett and Swim, 1998). That is, the difference in perceived discrimination might be partially attributable to differences in the accuracy of “discrimination judgments.” Individuals with a higher BMI appear to be aware of being liked or disliked, particularly when information about them circulates within their social network and provides them with relevant cues about their likeability. In comparison, individuals with a lower BMI do not seem to be aware whether they are liked or disliked even when others exchange gossip about them. Thus, taking a multi-informant approach to discrimination reveals that individuals with higher BMI form a realistic impression of their social inclusion, whereas individuals with lower BMI maintain a positive illusion about their social status.

However, why should individuals with higher BMI perceive discrimination more accurately? According to research on the process and accuracy of personality judgments, people learn relevant cues for personality traits, such as Extraversion, from experience (e.g., Funder, 1999; Hartung and Renner, 2011). Similarly, previous experience of discrimination might “teach” individuals relevant cues for discrimination (Feldman Barrett and Swim, 1998) and might render even the most minimal cues visible. Thus, overweight individuals may acquire more expertise and a heightened sensitivity to even subtle cues of discrimination in their social environment.

Interestingly, when not part of the social news and thereby deprived of relevant cues about their likeability, overweight individuals felt less socially included than non-overweight individuals. Research suggests that a history of discrimination or even simply knowing that one belongs to a stigmatized group (i.e., obese individuals) might establish a higher readiness to interpret ambiguous cues in the social environment as discriminatory (i.e., “zero-miss-strategy,” Feldman Barrett and Swim, 1998). Thus, in accordance with previous findings, these results suggest that the stigma of being overweight may affect the stigmatized individual even in the absence of actual discrimination (e.g., Steele and Aronson, 1995).

In combination with findings that “positive illusions” about the self have positive effects on psychological well-being (Taylor and Brown, 1988), one might speculate that the reduced psychological well-being of overweight individuals (Puhl and Heuer, 2009) is due on the one hand to more readily interpreting ambiguous situations as discriminatory and, on the other hand, a more accurate perception of their social environment.

Several limitations of the present research need to be acknowledged. Apart from the limitations that arise from the use of self-report measures and a non-representative sample (predominantly female and students), the study has limitations with regard to the generalization of the findings. In contrast to other discriminatory attributes, overweight is highly visible (Crocker et al., 1993), perceived as being at the responsibility of the individual (e.g., Weiner et al., 1988) and, additionally, no federal laws prohibit weight discrimination (e.g., Andreyeva et al., 2008; see also the German *General Equal Treatment Law*). Therefore, whether the present results can be generalized to invisible and/or uncontrollable discriminatory attributes remains open to further research. Another limitation is that the present study assessed gossip activity without taking the valence of the content into account. Since transmitted and perceived social information might affect actual and perceived social exclusion and discrimination differently, additional information regarding the content of gossip would be a promising avenue for future research.

## Conflict of Interest Statement

The authors declare that the research was conducted in the absence of any commercial or financial relationships that could be construed as a potential conflict of interest.
